# Frailty impacts all-cause mortality after endovascular abdominal aortic aneurysm repair: a retrospective cohort study

**DOI:** 10.1016/j.jnha.2025.100489

**Published:** 2025-01-18

**Authors:** Yi-Xuan Wang, Wen-Xin Zhao, Zi-Mo Wang, Ning Zhao, Zhao-long Li, Zhi-Yuan Wu, Yong-Peng Diao, Yong-Jun Li

**Affiliations:** a Department of Vascular Surgery, Beijing Hospital, National Center of Gerontology, Institute of Geriatric Medicine, Chinese Academy of Medical Sciences, Beijing, China; b Peking University Fifth School of Clinical Medicine; c Institute of Molecular Vascular Medicine, Technical University Munich, Munich, Germany; d German Center for Cardiovascular Research DZHK, Partner Site Munich Heart Alliance, Berlin, Germany

**Keywords:** Frailty, Frailty tools, Abdominal aortic aneurysm, Vascular surgery, Aging, Abdominal aortic aneurysm repair

## Abstract

**Objective:**

This study aimed to evaluate the impact of frailty and inflammation on all-cause mortality in patients with abdominal aortic aneurysm (AAA) who underwent endovascular aneurysm repair (EVAR), and key risk factors were also explored.

**Methods:**

A retrospective analysis was conducted on 174 patients with AAA who underwent EVAR at Beijing Hospital between 2016 and 2024. Frailty was assessed using the modified five-item Frailty Index (mFI-5). Inflammation was quantified by the red cell distribution width-to-albumin ratio (RAR), a novel inflammatory marker. We examined the associations between frailty, preoperative risk factors, and mortality using Kaplan-Meier survival analysis and Cox proportional hazards models. Mediation analysis was performed to evaluate the role of RAR in the relationship between frailty and mortality.

**Results:**

Frailty was found to be an independent risk factor for all-cause mortality following EVAR (HR = 1.95, P = 0.048). Preoperative anemia (HR = 0.98, P = 0.032), elevated creatinine levels (HR = 1.01, P = 0.013), and prolonged operation time (HR = 1.01, P = 0.029) were also independent predictors of mortality. Kaplan-Meier survival analysis revealed significantly lower survival rates for frailty patients (P = 0.004). Additionally, RAR mediated 23.8% of the relationship between frailty and mortality (P = 0.012), underscoring its role as a key indicator of chronic inflammation.

**Conclusions:**

Frailty and chronic inflammation, as measured by the innovative RAR marker, are significant contributors to mortality after EVAR. This study highlights the clinical utility of RAR in identifying high-risk AAA patients and its potential for guiding targeted preoperative interventions. Incorporating frailty assessments and inflammation monitoring into routine preoperative evaluations may improve patient outcomes by enabling personalized approaches such as nutritional optimization and inflammation control.

## 1 Introduction

Despite the advancements in endovascular aortic repair (EVAR) as the preferred treatment approach for abdominal aortic aneurysm (AAA) [1], postoperative outcomes are influenced by patient-related factors beyond anatomical considerations. How to evaluate the patient well before surgery has always been a difficult problem.

Frailty is a well-established independent risk factor for poor postoperative outcomes, particularly in elderly or high-risk patients. Frail individuals experience higher rates of mortality and complications after surgery [2]. The Modified Frailty Index-5 (mFI-5), a simplified frailty assessment tool, effectively predicts surgical outcomes [[2], [3], [4]], including 30-day mortality and complications in vascular procedures [5]. Additionally, the mFI-5 has been linked to 30-day mortality and complication rates following open abdominal aortic aneurysm repair [6]. However, limited research has examined frailty’s impact on all-cause mortality following EVAR using the mFI-5.

Frailty’s adverse effects may partly result from its association with inflammation, which plays a crucial role in postoperative stress responses [7]. Among various inflammatory markers, the red cell distribution width-to-albumin ratio (RAR) has emerged as a reliable predictor of outcomes in cardiovascular and surgical settings [8,9]. Compared with other markers such as C-reactive protein (CRP), neutrophil-to-lymphocyte ratio (NLR), or platelet-to-lymphocyte ratio (PLR), RAR uniquely integrates systemic inflammation and nutritional status. This study investigates whether frailty independently predicts mortality after EVAR and examines the role of RAR as a mediator in this relationship, hypothesizing that RAR reflects inflammation’s contribution to frailty-related mortality.

## 2 Study design and population

A retrospective analysis was conducted on patients diagnosed with AAA at Beijing Hospital from August 2016 to May 2024. The inclusion criteria were: (1) patients with AAA confirmed through imaging examinations such as CTA, DSA, or ultrasound; (2) patients undergoing their first EVAR procedure; (3) patients with complete perioperative and follow-up data; (4) patients who provided informed consent for the EVAR procedure after being informed about alternative treatment options; (5) the patient was older than 60 years (Supplementary Figure 1). The study was approved by the Institutional Review Board (approval number: 2023BJYEC-128-01).

## 3 Definitions and variables

Clinical data were retrieved from the database including baseline clinical characteristics, demographics, medical history, comorbidities, operation time, medication usage, aneurysm diameter, and laboratory data. Frailty was defined using the modified five-item Frailty Index (mFI-5) developed by the American College of Surgeons National Surgical Quality Improvement Program (ACS NSQIP) [10]. This tool incorporates both functional status and comorbidities, assigning 1 point for each of the following: congestive heart failure, chronic obstructive pulmonary disease (COPD) or recent pneumonia, hypertension requiring medication, diabetes mellitus, and non-independent functional status.

Comorbidity and functional status were retrospectively determined using preoperative anesthetic assessments and medical records. Functional status was evaluated using the ADL (Activities of Daily Living) score, obtained from the nursing care system. Frailty was defined as an mFI-5 score of ≥2 [11].

## 4 Statistical analysis

The Shapiro-Wilk test assessed the normality of continuous variables, presented as mean ± SD or median (IQR) depending on distribution. Categorical variables were expressed as counts and percentages. Missing data were imputed using the mean for normally distributed variables and the median or mode otherwise. Group comparisons utilized independent t-tests or Mann-Whitney U tests for continuous variables and chi-square or Fisher’s exact tests for categorical variables. Kaplan-Meier survival curves with log-rank tests compared mortality between frailty and non-frailty groups, and Cox regression identified mortality risk factors. Mediation analysis, performed with the mediator package, assessed frailty (exposure), RAR (mediator), and mortality (outcome). Path a examined frailty-RAR association, path b evaluated RAR-mortality association, and path c’ measured the direct frailty-mortality effect (Fig. 2). Mediated effects were calculated as (mediated effect/total effect) × 100% with bootstrapping for significance. Analyses were conducted in R 4.4.3 with P < 0.05 considered significant.

## 5 Results

### 5.1 Differences between dead and survival groups and between frailty and non-frailty groups

The median follow-up period was 25.93 ± 1.50 months (range: 0–69 months). During the follow-up, 45 patients (25.86%) died. Compared to survivors, patients in the death group were significantly older (P = 0.003) and had larger aneurysm diameters (P = 0.04). Diabetes was more prevalent in the death group (P = 0.011), while other comorbidities showed no significant differences.

Preoperative blood test results revealed that the death group had significantly lower hemoglobin (P < 0.001), albumin (P = 0.017), and lymphocyte count (P = 0.031), along with higher RAR (P = 0.005) (Supplementary Table 1).

Similarly, frailty patients (n = 57) were older (P = 0.003) and had higher mortality rates (P = 0.007) compared to non-frailty patients (n = 117). Frailty was also associated with increased rates of diabetes (P < 0.001), stroke (P = 0.048), and hypertension (P < 0.001) (Table 1A).

**Table 1:** Baseline characteristics and cox regression results.

(A) Baseline Patient Characteristics by Frailty				
Parameters	Total (174)	Non-frailty (117)	Frailty (57)	P value
**Demographic**				
Age, years	72.00 (67.00,79.00)	69.00 (67.00, 77.00)	75.00 (70.00, 81.00)	0.003*
Male, n (%)	154 (88.51)	105 (89.74)	49 (85.96)	0.463
BMI, kg/m^2^	24.25 ± 3.74	24.32 ± 3.66	24.09 ± 3.94	0.711
Death, n (%)	45 (25.86)	23 (19.66)	22 (38.60)	0.007*
**Medical history and comorbidities, n (%)**				
Smoking	86 (49.43)	58 (49.57)	28 (49.12)	0.956
Diabetes	32 (18.39)	6 (5.13)	26 (45.61)	<.001*
Hyperlipidemia	59 (33.91)	39 (33.33)	20 (35.09)	0.819
COPD	13 (7.47)	4 (3.42)	9 (15.79)	0.009*
Atrial fibrillation	11 (6.32)	8 (6.84)	3 (5.26)	0.945
Hypertension	127 (72.99)	73 (62.39)	54 (94.74)	<.001*
Aspirin	88 (50.57)	57 (48.72)	31 (54.39)	0.483
Stroke	34 (19.54)	18 (15.38)	16 (28.07)	0.048*
**Blood test**				
WBC count, (*10^9^/L)	6.20 (5.33, 7.63)	6.20 (5.46, 7.53)	6.16 (5.13, 7.74)	0.531
Platelet count, (*10^9^/L)	181.91 ± 62.79	182.38 ± 57.72	180.95 ± 72.65	0.888
Hemoglobin, g/L	128.50 (115.25,141.00)	133.00 (119.00,143.00)	119.00 (104.00,134.0)	<0.001*
Neutrophil count, (*10^9^/L)	3.84 (3.14, 4.84)	3.79 (3.15, 4.70)	4.02 (3.10, 5.03)	0.436
Lymphocyte count, (*10^9^/L)	1.53 (1.14, 2.05)	1.66 (1.23, 2.13)	1.25 (0.98, 1.73)	<0.001*
Monocyte count, (*10^9^/L)	0.47 (0.39, 0.56)	0.47 (0.39, 0.56)	0.48 (0.39, 0.56)	0.816
Creatinine, μmol/L	86.00 (73.25, 106.00)	85.00 (73.00, 106.00)	90.00 (75.00, 106.00)	0.621
Albumin, g/L	38.50 (36.00, 40.00)	39.00 (36.00, 40.00)	37.00 (35.00, 40.00)	0.066
RDW	13.00 (12.60, 13.80)	13.00 (12.50, 13.70)	13.10 (12.60, 14.00)	0.220
RAR	0.34 (0.32, 0.38)	0.33 (0.31, 0.37)	0.35 (0.32, 0.39)	0.049*
Aneurysm diameter, mm	55.00 (50.00, 64.00)	55.00 (50.00, 64.00)	55.00 (51.00, 65.00)	0.683
Operation time, min	105.50 (75.00, 165.00)	105.00 (75.00, 150.00)	120.00 (75.00,180.00)	0.218

(B) Cox regression analysis showed the independent predictive effects of frail and other factors on mortality				
Parameters	Univariable Analysis		Multivariable Analysis	
	HR (95%CI)	P value	HR (95%CI)	P value
Frailty	2.33 (1.28∼4.23)	0.005*	1.95 (1.01∼3.77)	0.048*
Operation time	1.01 (1.01∼1.01)	0.043*	1.01 (1.01∼1.01)	0.029*
Creatinine	1.01 (1.01∼1.01)	0.002*	1.01 (1.01∼1.01)	0.013*
Hemoglobin	0.98 (0.97∼0.99)	<.001*	0.98 (0.97∼0.99)	0.032*

WBC, White Blood Cell. RDW, Red Cell Distribution Width. COPD, Chronic Obstructive Pulmonary Disease.

RAR, RDW/Albumin.

### 5.2 Kaplan-Meier survival curves evaluating frailty status as a predictor of all-cause mortality after EVAR

**Fig. 1:**
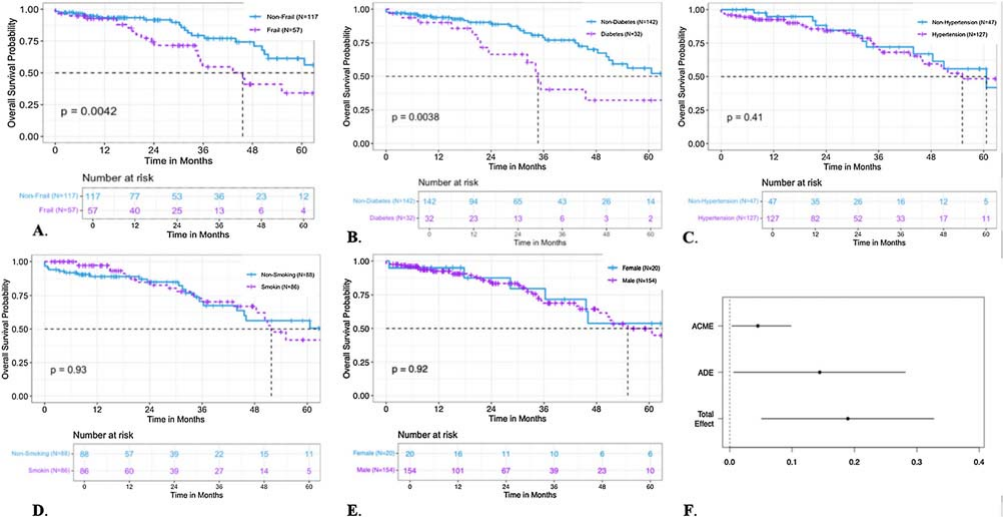
Kaplan–Meier Survival Curves and Causal Mediation Analysis. 1A, Kaplan–Meier Survival Curves Comparing Frailty and Non-Frailty Groups Post-EVAR, 1B, Kaplan–Meier Survival Curves Comparing Diabetes and Non-Diabetes Groups Post-EVAR, 1C, Kaplan–Meier Survival Curves Comparing Hypertension and Non-Hypertension Groups Post-EVAR, 1D, Kaplan–Meier Survival Curves Comparing Smoking and Non-Smoking Groups Post-EVAR, 1E, Kaplan–Meier Survival Curves Comparing Female and Male Groups Post-EVAR, 1F, Causal Mediation Analysis of RAR ACME, Average Causal Mediation Effect. ADE, Average Direct Effect.

Kaplan-Meier survival analysis showed that frailty patients had a median survival time of 45.60 ± 5.77 months, compared to 65.2 ± 3.70 months for non-frailty patients. The log-rank test indicated a significant difference in survival between the two groups (P = 0.004) **(**Fig. 1A**)**. In addition to our primary analysis, we performed subgroup analyses using Kaplan-Meier (KM) survival curves to compare post-EVAR survival outcomes across several patient groups. The specific subgroup comparisons included: hypertension, diabetes, gender, smoking history. These subgroup analyses aimed to explore potential differences in survival based on these clinically significant factors. The Kaplan-Meier curves for each comparison are presented in Fig. 1, illustrating cumulative survival probabilities over time post-EVAR.

**Fig. 2:**
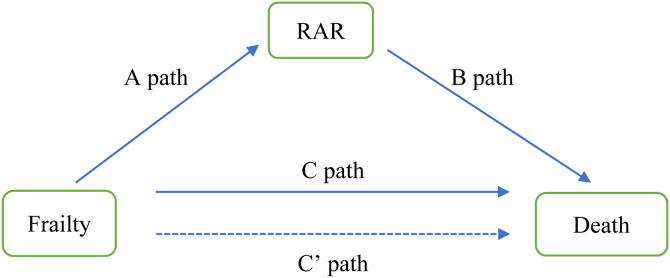
Path diagram of the mediation analysis models.

### 5.3 Univariate and multivariate cox regression analysis of the hazard ratio (HR) for death after elective EVAR

Univariate Cox regression analysis identified several factors significantly associated with mortality, including diabetes (HR 2.49 [1.31–4.72], P = 0.005), frailty (HR 2.33 [1.28–4.23], P = 0.005), age (HR 1.05 [1.01–1.09], P = 0.019), albumin (HR 0.86 [0.79–0.94], P < 0.01), hemoglobin (HR 0.98 [0.97–0.99], P < 0.001), creatinine (HR 1.01 [1.01–1.01], P = 0.002), and operation time (HR 1.01 [1.01–1.01], P = 0.043). In multivariate Cox regression, frailty (HR 1.95 [1.01-3.77], P = 0.048), operation time (HR 1.01 [1.01–1.01], P = 0.029), creatinine (HR 1.01 [1.01–1.01], P = 0.013), and hemoglobin (HR 0.98 [0.97–0.99], P = 0.032) remained independent risk factors for mortality (Table 1B).

### 5.4 Subgroup interaction and Analysis of mediation

Subgroup analysis revealed no significant interaction effects between frailty and mortality across various subgroups (Table 2A). Mediation analysis showed that inflammatory status mediated the relationship between frailty and mortality, with a significant average causal mediation effect (P = 0.01) and a total effect (P = 0.002) (Table 2B). The Proportion Mediated ratio was 23.8% (P = 0.012). This suggests that frailty may be mediated by inflammation (Fig. 1F).

**Table 2:** Subgroup analysis and Causal Mediation Analysis.

A Subgroup analyses were performed for sex, diabetes, smoking history, and hypertension.			
Parameters	HR (95%CI)	P value	P for interaction
Sex			0.981
Male	2.302 (1.200∼4.4145)	0.012	
Female	2.721 (0.495∼14.946)	0.250	
Diabetes			0.998
Yes	1.842 (0.857∼3.955)	0.118	
No	1.818 (0.401∼8.244)	0.438	
Smoking			0.222
Yes	3.519 (1.479∼8.378)	0.004	
No	1.517 (0.645∼3.567)	0.339	
Hypertension			0.803
Yes	2.397 (1.163∼4.941)	0.018	
No	2.792 (0.599∼13.006)	0.190	

B Causal mediation analysis.				
	Estimate	95% CI Lower	95% CI Upper	P value
ACME	0.04	0.00743	0.10	0.010*
ADE	0.14	0.00589	0.28	0.038 *
Total Effect	0.19	0.04931	0.33	0.002 *
Prop. Mediated	0.23	0.03976	0.86	0.012 *

ACEM, Average Causal Mediation Effect. ADE, Average Direct Effect.

## 6 Discussion

In this retrospective study, several key findings were identified: (1) frailty is an independent risk factor for all-cause mortality following EVAR. (2) Preoperative anemia, elevated creatinine levels, and prolonged operation time were all confirmed as independent predictors of postoperative all-cause mortality. (3) An inflammatory marker, RAR, mediated the relationship between frailty and mortality, accounting for approximately 23.8% of the mortality risk.

Previous studies have defined frailty as a clinical syndrome characterized by a persistent loss of internal homeostasis, leading to increased vulnerability to stressors, particularly in elderly individuals [[12], [13], [14]]. Our findings provide new insights, indicating that frailty is associated with increased all-cause mortality following EVAR, potentially due to chronic inflammation. Specifically, frail patients tend to have elevated RAR, which may reflect the dysregulation of systemic inflammatory processes and contribute to their higher risk of adverse postoperative outcomes. In these patients, chronic inflammation, as indicated by elevated RAR, may impair the body’s ability to respond effectively to surgical stressors, further increasing the likelihood of complications and mortality.

RAR, which reflects changes in red cell distribution width and serum albumin levels, is closely linked to systemic inflammation. In frail patients, an imbalance in inflammatory pathways may exacerbate the body’s stress response to surgery, leading to worse outcomes. In response to postoperative stressors, frailty patients may face an elevated risk of mortality due to dysregulated inflammatory responses. These findings have significant clinical implications, particularly for the preoperative evaluation and postoperative management of elderly patients undergoing EVAR. Improvement in Preoperative Assessment: Our results support the routine use of the mFI-5 as a simple yet effective tool to assess frailty in elderly EVAR patients. By identifying frailty as an independent risk factor for all-cause mortality, our study underscores the importance of incorporating frailty assessments into preoperative evaluations. This enables early identification of high-risk patients, allowing clinicians to customize surgical strategies and postoperative management plans to mitigate frailty-related risks.

These conclusions are consistent with previous research, which suggests that frailty patients may benefit from preoperative interventions such as nutritional optimization, physiotherapy, and more intensive perioperative monitoring [15]. Importance of Inflammation Monitoring: RAR was found to mediate the relationship between frailty and mortality, accounting for 23.8% of the mortality risk. This highlights RAR’s potential as a predictive marker for postoperative outcomes in EVAR patients. Regular monitoring of RAR and other inflammatory markers, both preoperatively and postoperatively, could aid in the early detection of high-risk patients and inform targeted interventions. Dysregulated inflammation could exacerbate frailty and compromise recovery, making RAR a valuable biomarker for identifying individuals at risk. Importantly, the applicability of RAR as a marker for other types of surgical patients requires further investigation, as it may offer broader clinical utility beyond EVAR. Implementing early anti-inflammatory strategies or treatments aimed at controlling inflammation may improve recovery and reduce mortality rates in frailty patients.

Preoperative anemia is an independent risk factor for postoperative all-cause mortality, especially in elderly patients. Aging is associated with diminished immune function and increased systemic inflammation, both of which contribute to the high prevalence of anemia in older adults. This interplay between aging, inflammation, and anemia may worsen health outcomes, increasing frailty and vulnerability to surgical complications [[16], [17], [18], [19]]. With aging, the immune system’s capacity to control and respond to inflammation declines, leading to increased systemic inflammation, which can significantly worsen health outcomes and reduce overall quality of life, making elderly individuals more vulnerable to conditions such as anemia [20,21]. Consequently, age-related chronic inflammation is not merely a byproduct of aging; it is also considered an important marker of biological aging, multimorbidity, and increased mortality risk [22]. Studies have shown that anemia is not only closely related to inflammation and aging but also significantly impacts the surgical prognosis of AAA patients. Nicolas et al. found that hemoglobin concentration is independently associated with AAA size and reduced long-term survival following EVAR [23]. Additionally, a study by Dakour-Aridi et al., published in 2018, concluded that preoperative anemia is associated with higher odds of 30-day mortality and major in-hospital adverse outcomes after EVAR [24]. Our findings align with these studies, supporting that preoperative anemia is indeed a reliable predictor of all-cause mortality following EVAR.

Serum creatinine levels and prolonged operation time were also identified as independent risk factors for mortality. Elevated creatinine levels may indicate impaired renal function or an increased risk of postoperative acute kidney injury, which has been linked to higher mortality rates [25]. While prolonged operative time during EVAR can increase exposure to contrast agents and radiation, both of which heighten the risk of postoperative complications [26,27]. These findings underscore the importance of minimizing operative time and monitoring renal function in order to reduce the risk of adverse outcomes.

This study has several limitations. First, as a retrospective analysis, it inherently includes potential biases, such as selection bias and data bias. Although we included patients who met the inclusion and exclusion criteria, the retrospective nature of this study limits our ability to establish causal relationships between frailty, inflammatory markers, and mortality. Second, our study was conducted at a single center with a relatively small sample size, which may limit the generalizability and statistical power of the findings. Patient characteristics and treatment protocols may vary across different institutions, affecting the reproducibility of our results. A multicenter study with a larger sample size would help to validate and strengthen these findings. Additionally, while the measurement methods for mFI-5 and RAR were well-defined and evidence-based, they have inherent limitations. The mFI-5, as a simplified frailty assessment tool, may not capture all dimensions of frailty, potentially underestimating its complexity. Similarly, RAR, while a reliable marker of systemic inflammation, may be influenced by factors such as acute infections or nutritional status, which could confound its interpretation. Future research should consider incorporating more comprehensive frailty assessments and exploring additional inflammatory markers to validate and expand upon our findings.

## 7 Conclusion

Frailty is a significant predictor of mortality after EVAR, and chronic inflammation, as indicated by RAR, mediates part of this effect. Our findings highlight the importance of incorporating frailty assessments and inflammation monitoring into the preoperative evaluation of AAA patients to better identify high-risk individuals. Targeted preoperative interventions, such as nutritional optimization and inflammation control, may help improve postoperative outcomes and reduce mortality in frailty patients.

## Funding

This research was funded by the National High Level Hospital Clinical Research Funding Project (No. BJ-2024-142)

## Declaration of competing interest

The authors declare that there are no financial or other interests that could be perceived as conflicts of interest regarding the research and publication of this study. All authors confirm their contributions to the work and agree with the publication of the manuscript. The study was independently conducted without any external funding that could influence the results or interpretation of the findings.
