# Supplementary material for: Frailty impacts all-cause mortality after endovascular abdominal aortic aneurysm repair: a retrospective cohort study
Source: J Nutr Health Aging. 2025 Jan 18;29(4):100489. doi: 10.1016/j.jnha.2025.100489 (PMC12180044; doi:10.1016/j.jnha.2025.100489)
Supplement: Supplementary file 1 [file mmc1.docx]

Table 1 Baseline Patient Characteristics by Group

| **Parameters** | **Total**  **(174)** | | **Survival**  **(129)** | | **Death**  **(45)** | | **P value** | | |  |
| --- | --- | --- | --- | --- | --- | --- | --- | --- | --- | --- |
| **Demographic** | | | | | | | | |  |  |
| Age, years | 72.00(67.00,79.00) | | 71.00(67.00,78.00) | | 76.00(69.00,81.00) | | **0.003*** | | |  |
| Male, n (%) | 154 (88.51) | | 117 (90.70) | | 37 (82.22) | | 0.125 | | |  |
| BMI, kg/m^2^ | 24.25 ± 3.74 | | 24.27 ± 3.67 | | 24.17 ± 3.98 | | 0.944 | | |  |
| Frailty, n (%) | 117 (67.24) | | 94 (72.87) | | 23 (51.11) | | **0.007*** | | |  |
| Aneurysm diameter, mm | 55.00 (50.00, 64.00) | | 55.00 (50.00, 61.00) | | 59.00 (50.00, 71.00) | | **0.04*** | | |  |
| Operation time, min | 105.50 (75.00, 165.00) | | 105.00 (75.00, 150.00) | | 115.00 (88.00, 180.00) | | 0.182 | | |  |
| **Medical history and comorbidities, n (%)** | | | | | | | | |  |  |
| Smoking | 86 (49.43) | | 64 (49.61) | | 22 (48.89) | | 0.933 | | |  |
| Diabetes | 32 (18.39) | | 18 (13.95) | | 14 (31.11) | | **0.011*** | | |  |
| Hyperlipidemia | 59 (33.91) | | 45 (34.88) | | 14 (31.11) | | 0.645 | | |  |
| COPD | 13 (7.47) | | 9 (6.98) | | 4 (8.89) | | 0.928 | | |  |
| Atrial fibrillation | 11 (6.32) | | 8 (6.20) | | 3 (6.67) | | 1.000 | | |  |
| Hypertension | 127 (72.99) | | 94 (72.87) | | 33 (73.33) | | 0.952 | | |  |
| Aspirin | 88 (50.57) | | 61 (47.29) | | 27 (60.00) | | 0.142 | | |  |
| Stroke | 34 (19.54) | | 21 (16.28) | | 13 (28.89) | | 0.066 | | |  |
| **Blood test** | | | | | | | | |  |  |
| WBC count, (*10^9^/L) | 6.20 (5.33, 7.63) | | 6.07 (5.27, 7.53) | | 6.56 (5.42, 8.00) | | 0.375 | | |  |
| Platelet count, (*10^9^/L) | 181.91 ± 62.79 | | 183.81 ± 56.75 | | 176.47 ± 78.06 | | 0.411 | | |  |
| Hemoglobin, g/L | 128.50(115.25,141.00) | | 133.00(119.00,143.00) | | 118.00(102.00,129.00) | | **<.001*** | | |  |
| Neutrophil count, (*10^9^/L) | | 3.84 (3.14, 4.84) | | 3.79 (3.16, 4.69) | | 4.20 (2.92, 5.23) | | 0.365 | | |
| Lymphocyte count, (*10^9^/L) | 1.53 (1.14, 2.05) | | 1.60 (1.15, 2.16) | | 1.32 (1.12, 1.80) | | **0.031*** | | |  |
| Monocyte count, (*10^9^/L) | 0.47 (0.39, 0.56) | | 0.47 (0.39, 0.55) | | 0.49 (0.40, 0.57) | | 0.186 | | |  |
| Creatinine, μmol/L | 86.00 (73.25, 106.00) | | 84.00 (74.00, 103.00) | | 92.00 (73.00, 124.00) | | 0.08 | | |  |
| Albumin, g/L | 38.50 (36.00, 40.00) | | 39.00 (36.00, 41.00) | | 37.00 (35.00, 39.00) | | **0.017*** | | |  |
| RDW | 13.00 (12.60, 13.80) | | 13.00 (12.50, 13.70) | | 13.10 (12.70, 14.60) | | 0.06 | | |  |
| RAR | 0.34 (0.32, 0.38) | | 0.33 (0.31, 0.37) | | 0.35 (0.33, 0.41) | | **0.005*** | | |  |

WBC, white blood cell. RDW, red cell distribution width. COPD, Chronic obstructive pulmonary disease

RAR, RDW/ Albumin.

Figure 1 Flow diagram

Systematic registered for patients who underwent EVAR procedure

from 08/2016 to 05/2024

(n=202)

22 excluded:

- Open surgery (n = 13)
- Age <60 (n = 8)
- Thoracic aortic aneurysm （n=1）

Procedures with eligibility

(n=180)

6 without follow-up information

Data available for final analysis

(n=174)
